# Structural Studies on the Shapeshifting Murine Norovirus

**DOI:** 10.3390/v13112162

**Published:** 2021-10-26

**Authors:** Michael B. Sherman, Alexis N. Williams, Hong Q. Smith, B. Montgomery Pettitt, Christiane E. Wobus, Thomas J. Smith

**Affiliations:** 1 Department of Biochemistry and Molecular Biology, University of Texas Medical Branch at Galveston, 301 University Boulevard, 5.104D Basic Science Building, Route 0645, Galveston, TX 77555, USA; mbsherma@utmb.edu (M.B.S.); alnwilli@UTMB.EDU (A.N.W.); hqsmith@utmb.edu (H.Q.S.); mpettitt@utmb.edu (B.M.P.); 2 Department of Microbiology and Immunology, University of Michigan Medical School, 1150 West Medical Center Dr., Ann Arbor, MI 48109, USA; cwobus@umich.edu

**Keywords:** norovirus, antibodies, bile, neutralization

## Abstract

Noroviruses are responsible for almost a fifth of all cases of gastroenteritis worldwide. The calicivirus capsid is composed of 180 copies of VP1 with a molecular weight of ~58 kDa. This coat protein is divided into the N-terminus (N), the shell (S) and C-terminal protruding (P) domains. The S domain forms a shell around the viral RNA genome, while the P domains dimerize to form protrusions on the capsid surface. The P domain is subdivided into P1 and P2 subdomains, with the latter containing the binding sites for cellular receptors and neutralizing antibodies. Reviewed here are studies on murine norovirus (MNV) showing that the capsid responds to several physiologically relevant cues; bile, pH, Mg^2+^, and Ca^2+^. In the initial site of infection, the intestinal tract, high bile and metal concentrations and low pH cause two significant conformational changes: (1) the P domain contracts onto the shell domain and (2) several conformational changes within the P domain lead to enhanced receptor binding while blocking antibody neutralization. In contrast, the pH is neutral, and the concentrations of bile and metals are low in the serum. Under these conditions, the loops at the tip of the P domain are in the open conformation with the P domain floating on a linker or tether above the shell. This conformational state favors antibody binding but reduces interactions with the receptor. In this way, MNV uses metabolites and environmental cues in the intestine to optimize cellular attachment and escape antibody binding but presents a wholly different structure to the immune system in the serum. To our knowledge, this is the first example of a virus shapeshifting in this manner to escape the immune response.

## 1. Introduction

There are 11 genera in the *Caliciviridae* family of which seven infect animals including noroviruses. Noroviruses are further divided into 10 genogroups (GI-GX) that are further subdivided into 49 genotypes: 9 GI, 27 GII, 3 GIII, 2 GIV, 2 GV, 2 GVI and 1 genotype each for GVII, GVIII, GIX (formerly GII.15) and GX [1]. Noroviruses are the major cause of epidemic gastroenteritis in humans (for review, see [2]), causing ~20 million cases annually, resulting in more than 70,000 hospitalizations and 570–800 deaths in the US alone. While not often a fatal disease in the developed world, norovirus infections are estimated to cost more than USD 2 billion per year for healthcare and lost productivity. Controlling the spread of norovirus is challenging since as few as ten virions are sufficient to infect an adult [3].

Efforts to make effective norovirus vaccines have been thwarted by our lack of understanding of the structural mechanisms of viral escape from antibody neutralization. In addition, noroviruses are constantly evolving and frequently generating new strains [4,5,6] that result in worldwide epidemics [6,7]. Developing efficacious vaccines requires a detailed understanding of how escape mutations block antibody binding and thereby evade the immune system. While there have been advances in cell culture methods for the human noroviruses [8,9], the lack of small animal models has made in-vivo analyses more difficult [10]. Murine norovirus (MNV-1, genotype GV.1) is a powerful surrogate for the human viruses since it can be grown to high titers in cell culture, there is a reverse genetic system, and mice serve as a convenient animal model system.

**Figure 1:**
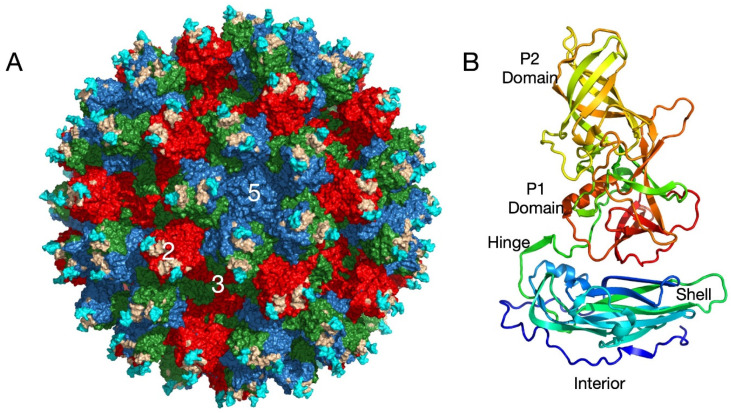
Overall architecture of the caliciviruses. (**A**) This figure shows the entire capsid of murine norovirus (MNV) observed in the cryo-EM structures in the presence of bile [11] and at low pH [20]. The subunits A, B, and C are shown in blue, green, and red, respectively. The P domain dimers are composed of A and B subunits around the 5-fold axes and of C dimers at the 2-fold axes. Also highlighted are the A’-B’ (cyan) and E’-F’ (tan) loops discussed in the text. (**B**) Shown here is one copy of the capsid protein colored from blue to red as the chain extends from the amino to carboxyl termini. Note that this represents the contracted form of the capsid seen at low pH [20] or in the presence of bile [11].

Caliciviruses are T = 3 icosahedral particles (Figure 1) with 180 copies of the major capsid protein (VP1; ~58 kDa), which is divided into the N-terminus (N), the shell (S) and C-terminal protruding (P) domains [11,12,13,14,15]. The S domain forms a shell around the viral RNA genome and the P domains form protrusions comprised of A/B and C/C dimers. The P domain is further subdivided into P1 and P2 subdomains, with the latter containing the binding sites for cellular receptors [16,17] and neutralizing antibodies [1,18,19]. The overall architecture of murine norovirus is shown in Figure 1A with the three copies of VP1 in the icosahedral asymmetric unit being designated as subunits A (blue), B (green), and C (red). Also noted in this figure is the location of the A’-B’ (cyan) and E’-F’ loops (tan) in the P2 domain that are discussed below. Figure 1B shows the structure of a single subunit [11] colored from blue to red as the chain extends from the amino to carboxyl termini. Note that this is the structure of MNV in the presence of bile [11] or at low pH [20] and is in the ‘contracted’ conformation with the P domain resting on the shell as discussed below.

The purpose of this review is to examine recent results demonstrating that the capsid of murine norovirus, and likely all noroviruses, is a dynamic structure that responds to several environmental/physiological cues. Under conditions found in the serum, the P domain rises more than 15 Å off the surface of the shell and is highly mobile. At the tip of the P domain are loops splayed apart in an ‘open’ conformation to which neutralizing antibodies bind. This all changes in the gut where high bile concentrations, low pH, and high Mg^2+^ and Ca^2+^ concentrations cause the P domains to rotate ~90° and contract onto the viral shell. Within the P domain, these conditions make the loops at the tip of the P domain convert to the ‘closed’ conformation, which blocks antibody binding while enhancing receptor binding. The results reviewed here strongly suggest that MNV escapes the immune system by shapeshifting from a contracted structure in the gut that is primed for infection to an open structure in the serum that elicits the immune response.

## 2. MNV Cell Receptor

Using CRISPR-Cas9 technology, a gene was identified that encodes a cell-surface protein, *Cd300lf* [21,22]. CD300lf is a member of the immunoglobin superfamily and is found on myeloid cells. This cell membrane protein has an extracellular domain of ~170 amino acids. Knocking out Cd300lf in murine macrophage BV2 cells blocked infection by MNV. Further, treating the cells with antibodies to CD300lf blocked MNV attachment. Even stronger evidence of the importance of CD300lf came from the demonstration that expression of this murine cell surface protein in HeLa cells made these human cells susceptible to MNV infection [22]. Further, they found that the bile acid glycochenodeoxycholic acid (GCDCA) enhances viral attachment to BV2 cells [23]. This is clearly a specific interaction since a chemically similar bile salt, taurocholic acid (TCA), had no effect on binding. These results were further substantiated using isothermal titration calorimetry that showed GCDCA binds to the expressed form of the P domain with Kd of ~6 µM, but TCA binding was too weak to detect [23]. Unlike human norovirus, MNV does not appear to interact with sialoglycans [24] and therefore the virus/host interactions differ significantly.

Using these soluble forms of CD300lf and the P domains, Nelson et al. also directly measured the interaction affinity using surface plasmon resonance [23]. The monomeric CD300lf protein bound to the P domain with a Kd of ~219 µM. While this represents a very weak interaction, the other members of the CD300 family, who failed to confer susceptibility to MNV infection (murine CD300ld, murine CD300lh, and human CD300f), showed little to no binding. By adding calcium, the affinity improved to ~25 µM, and when GCDCA and calcium were both added, the affinity improved to ~12 µM. The neutralizing antibody, A6.2, was able to block binding of CD300lf, suggesting that at least in-vitro antibody neutralization is due to abrogation of receptor attachment.

## 3. Two Modes of Flexibility

MNV is a versatile system for which all the virological tools have been developed. MNV can be propagated in a cell culture system, pathogenesis and the host immune response can be examined in the murine model, large amounts of virus can be readily produced, neutralizing monoclonal antibodies have been isolated, and an infectious clone has been developed [25]. Therefore, the structure of MNV-1 was needed to inform further virological studies. Surprisingly, even at relatively low resolution, it was apparent that the structure of MNV-1 was significantly different from the Norwalk virus (NV) virus-like particle (VLP) crystal structure [12,26]. While the P domains of NV VLPs in the crystal structure rest upon the shell domain, MNV-1 from cryo-EM had a large gap in the density between the shell and protruding domains. This gives the appearance that the MNV-1 P domains lift off the shell (S domains) to form a second proteinaceous layer.

Since this unusual ‘floating P domain’ conformation was so different from the crystal structure of NV, the cryo-EM structure of another calicivirus, rabbit hemorrhagic disease virus (RHDV), VLP was also determined [26]. The structure of RHDV, genus Lagovirus, is somewhat between what was observed for MNV-1 and NV. Similar to MNV, the P domains are lifted off the surface of the shell, but have a different orientation compared to MNV. This orientation places the bottom edge of the A subunit P1 domain near the S domain near the 5-fold axes, causing false connectivity at lower contouring. Similar to MNV-1, the density of the shell is more defined than the P domains, suggesting a high degree of flexibility of the P domains. The density of the C/C dimers in RHDV are far more diffuse than that observed with the A/B dimers. It was therefore clear that even this different genus of *Caliciviridae* is like MNV with highly flexible P domains that do not contact the shell surface.

Since neither MNV nor RHDV infect humans, it was important to revisit the cryo-EM structure of a human norovirus. The human norovirus Vietnam026 (GII.10) at ~10 Å resolution appeared very similar to MNV and RHDV [27]. As with MNV and RHDV, the P domain appeared as a second outer shell, and the P domain was raised off the S domain by ~15 Å. Importantly, Hansman et al. also showed that this apparent P domain flexibility may play an important role in antibody binding. They determined the crystal structure of the P domain complexed with the Fab fragment from an antibody (5B18) that broadly recognized several different GII viruses and showed that the 5B18 Fab bound to a conserved region of the protruding domain [27]. This binding site is involved in interactions with other regions of the capsid and partially buried in the virus particle in the ‘contracted’ state where the P domain sits on top of the shell as in the NV crystal structure. Despite the occluded nature of the recognized epitope in the VLP structure, ELISA binding indicated that the 5B18 antibody was able to capture intact VLPs. The base of the P domain is only exposed to the antibody if there is extreme flexibility in the tether region between the shell and the P domain. This result has been further substantiated by latter studies with genotypes GI.1 [28] and GII.4 [29] where various epitopes are clearly buried in the contracted particle and only exposed if the P domain is allowed to lift off the shell. It should be noted that a more recent study by the same group [30] suggested some significant differences to their previous Hanson et al. publication as well as the MNV-1 studies described here. They suggested that the only way to see a ‘floating’ P domain in these viruses is to add EDTA in high pH buffers. A careful examination of their protocol suggests that, since the band of virus from the CsCl gradient was not dialyzed prior to pelleting, their deviation from other published results was due to metal contamination. Indeed, the extended conformation was always observed in neutral pH PBS buffers when the virus was purified using sucrose gradients (or dialyzed extensively whenever CsCl gradients were used) and EDTA was never added to the virus at any stage of the preparation. Nevertheless, the more important observation from the Song et al. studies is that this same floating/contraction phenomenon was also observed in human norovirus GII.3. Finally, we are observing the same phenomena in the WU23 strain (unpublished data) as described below for MNV-1. Therefore, this gross P domain movement is highly conserved, but its biological function remains uncertain.

**Figure 2:**
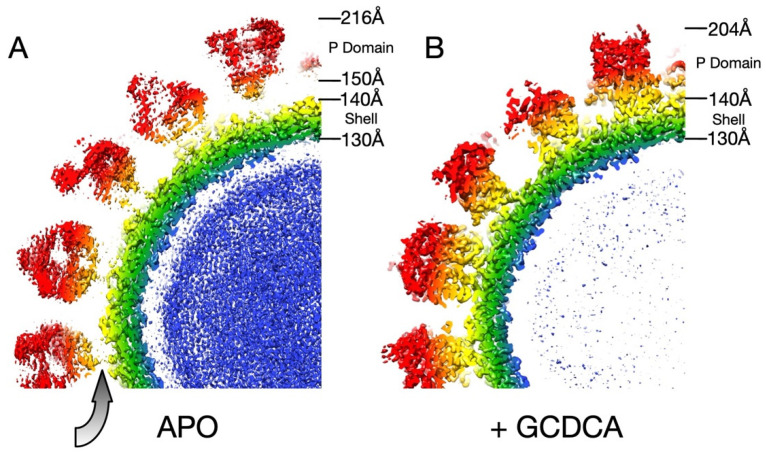
Cross sections of MNV-1 at pH 7.5 in the absence and presence of GCDCA [11]. Shown here are the central sections of the cryo-EM image reconstructions of MNV-1 in the absence (**A**) and presence (**B**) of GCDCA. The density surfaces are colored according to the distance from the center of the particle. Note that the P domain in the apo structure is more diffuse, suggesting mobility, and is >15 Å above the shell surface compared to the structure in the presence of GCDCA. The contracted structure observed in the presence of GCDCA was essentially identical to MNV-1 at pH 5.0 [20].

At the time of the earlier MNV-1 studies, it was not at all clear whether the P domains of the noroviruses could transition between this extended, flexible state [12,30] and the contracted state observed in NV [12,30]. This was indeed found to be the case with MNV. With the receptor identified, the next step was to determine the structure of the MNV/CD300lf complex. However, after several attempts to determine the cryo-EM structure of the complex, the bound receptor was never visible. When it was discovered that bile salts enhance receptor attachment [23], GCDCA was first added to MNV alone as a control. Using the same preparation of virus, high-resolution EM data were collected +/− GCDCA. As observed previously [12,31] when MNV is in PBS at pH 7.5, the P domain ‘floats’ by more than 15 Å above the surface of the shell domain. While the resolution of the apo reconstruction was calculated to be 3.1 Å, the density of the P domain was largely disordered. In contrast, when GCDCA was added, the P domain collapses down onto the shell surface and becomes well-ordered except for the outermost loops at the very tip of the P domain (Figure 2). Therefore, the addition of bile increases the affinity of the receptor to the virion and at the same time causes the contraction of the P domain onto the shell. From the cryo-EM structure of the MNV/CD300lf complex and from modeling of the complex onto the ‘floating P domain’ MNV structure, it appeared that the contracted structure of MNV had room for more copies of CD300lf to bind. In the expanded form, the C-termini of the CD300lf clash as trimers in the icosahedron, and therefore, there is not enough room for all the P domains to be saturated with receptors [11]. In contrast, the contracted conformation created more space around each of the P domains for a higher degree of saturation with the receptor. Interestingly, the P domains around the 5-fold axes are oriented such that there is clearly room for saturation, and it may be that icosahedral pentons play a large role in attachment. It is important to note that the flexibility of the linker likely allows for more conformational states than the two shown here.

It is also possible that the flexibility of the P domain plays a different role apart from affecting cell attachment. The flexible tether between the shell and the P domains could be sensitive to proteases that would release soluble P domains. This might act as an immune ‘smoke screen’ by presenting antigenic sites not well exposed on the viral surface. Indeed, P domains have been found in stool from NV infected volunteers [32], suggesting such a mechanism maybe relevant to human noroviruses as well. Similarly, the marked flexibility of the P domains in the expanded state might expose these antigenic sites while still attached to the shell. In this way, the immune response might focus on producing antibodies that bind poorly or not at all to the contracted capsid. This hypothesis is supported by the fact that some MNV antibodies were found to recognize buried epitopes on the shell domain [33], but none were neutralizing and were essentially immunological ‘dead ends.’ Similar results were found with human genotypes GII.10 [27], GI.1 [28], and GII.4 [29]. In essence, the highly flexible nature of the P domain could be a ‘moving target’ for the immune response.

### 3.1. MNV/Antibody Complexes

In our initial studies to understand how noroviruses might use P domain flexibility to escape antibody neutralization, we determined the cryo-EM structure of MNV complexed with the Fab fragment from a neutralizing A6.2 [12,26] and the crystal structure of Fab A6.2 [34]. From the EM structures, it was clear that A6.2 bound to the outermost tip of the P2 domain at the A’B’ and E’F’ loops. Typically, antibodies use the third hypervariable loop (CDR3) of the heavy chain to make the majority of the contacts with the epitope of the antigen [35]. Interestingly, this loop in A6.2 is strongly hydrophobic with the sequence ‘YFYALDYW’. When the crystal structures of A6.2 and the P domain dimer were placed into the cryo-EM density of the complex, it was evident that A6.2 fit better onto the open conformation with the hydrophobic CDR3 loop extending into the hydrophobic interface between the A’-B’ and E’-F’ loops [26]. In the closed conformation, these nonpolar residues in the P domain are deeply buried under the tips of the A’B’ and E’F’ loops and not accessible to mAb A6.2. In addition, the CDR3 loop of modeled A6.2 completely clashes with the E’-F’ loops in the closed conformation. Taken together, even at this relatively low resolution, the modeling strongly suggested that mAb A6.2 is only able to bind to the open conformation.

Nearly identical results were observed with a second neutralizing antibody, 2D3 [36]. Compared to A6.2, MNV-1 had far more difficulty in overcoming neutralization to 2D3, as it took over 20 passages for MNV-1 to escape mAb 2D3 neutralization [33,34]. The escape mutants to A6.2 were still neutralized by 2D3 and visa-versa. Importantly, all the MNV strains tested were neutralized to at least some degree by 2D3 while A6.2 was far more selective. This suggested that the epitope on the P domain region recognized by 2D3 was significantly different and more conserved than that for A6.2 and would therefore make a good vaccine target. Surprisingly, when the cryo-EM structure of the 2D3 bound to MNV was determined [33], it bound in nearly an identical location. Similar to A6.2, 2D3 appears to bind to the open but not the closed conformation of the P domain but mAb 2D3 appears to bind slightly deeper in the crevice between the A’-B’ and the E’-F’ loops. Surprisingly, neither of the natural escape mutants to 2D3 (D348E and V339I) are in contact with the bound antibody. It is highly unusual that none of the possible mutations at the paratope/epitope interface produced a viable virus. Therefore, both antibodies only bind to the open conformation but the escape mutations to 2D3 appear to act in an ‘allosteric’ manner.

**Figure 3:**
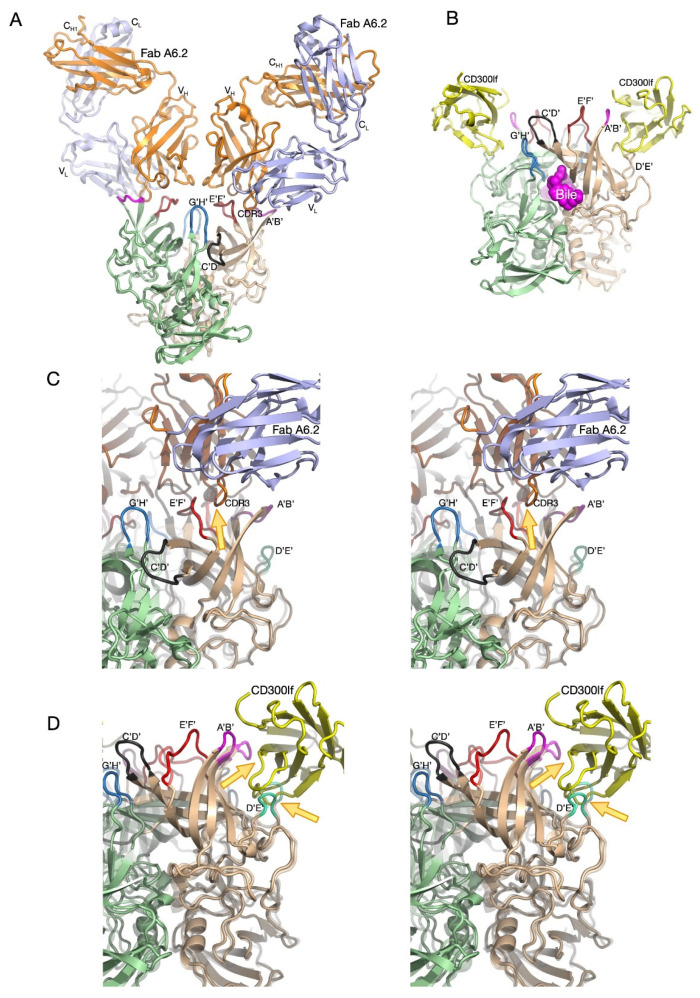
High-resolution structures of MNV-1 P domain complexed with a neutralizing antibody (A6.2) and the receptor CD300lf. (**A**) The ~3 Å cryo-EM structure of the isolated P domain complexed with neutralizing Fab A6.2 [37]. The two P domains are colored tan and light green and the antibody heavy and light chains are colored orange and light blue, respectively. The loops at the tip of the P domain, A’B’, C’D’, E’F’, and G’H’, are colored mauve, red, black, and blue, respectively. Note that the P domain is in the open conformation and that the CDR3 loop of the antibody reaches down between the A’B’ and E’F’ loops. (**B**) The crystal structure of the P domain complexed with GCDCA and the receptor CD300lf [23]. The color scheme of the P domain is the same as in (**A**) and CD300lf is shown in yellow. (**C**) Stereo figure of the Fab A6.2 bound to the open conformation of the P domain. The transparent image is the structure of the P domain in the closed conformation. The yellow arrow highlights the structural clashes between Fab A6.2 and the closed P domain structure. (**D**) Stereo figure of the P domain/CD300lf complex. With the transparent ribbon diagram of the P domain from the Fab complex.

The relatively low-resolution cryo-EM structures of the MNV virion complexed with Fab fragments [12] left several unanswered questions about the mechanism of neutralization. The most important question was whether it did indeed bind to the open conformation. To improve the resolution of our Fab/MNV whole virion structure, we initially attempted to use the fact that bile causes contraction of the P domain onto the shell that results in high-resolution density for the entire P domain. However, after several attempts, it was clear that the Fab was unable to bind to the MNV/bile complex. In hindsight, this is entirely because the Fab only recognizes the open conformation while bile induces the closed conformation. Therefore, we determined the near atomic cryo-EM structure (Figure 3A,C) of the isolated P domain complexed with Fab A6.2 [37]. As was observed in the pseudo-atomic model of intact MNV complexed with Fab A6.2 [12], one Fab is bound to each copy of the P domain protein and forms a ‘Y’ structure. While the density for most of the complex was clear and allowed for unambiguous interpretation, the density of the constant domains was more diffuse due to the marked flexibility of the Fab at the elbow region. Similar results were observed in the crystal structure of intact human rhinovirus 14 (HRV14) complexed with neutralizing Fab17 [38].

As was predicted from the previous pseudo-atomic structure of the MNV/A6.2 complex [12,26], the A’B’/E’F’ loops of the P domain are in the open conformation and the hydrophobic CDR3 loop of the antibody reaches down into the interface between the two loops. The positioning of A6.2 in that structure [12,31] was very close to that shown in Figure 3 but is rotated slightly such that fewer light chain contacts are made. As discussed below, the previous crystal structure of the A’B’/E’F’ loops in the open conformation [31] is only slightly different than the actual structure of the complex. Therefore, A6.2 is not inducing conformational changes in the P domain per se, but rather binding to a selected structure found in solution. In contrast, the hydrophobic residues in the heavy chain CDR3 loop in the crystal structure of Fab A6.2 alone [34] are folded upon themselves to avoid the aqueous environment. When A6.2 binds to the P domain, the CDR3 loop unfurls to meet the hydrophobic residues in the A’B’/E’F’ cleft. This is similar to the crystal structure of the HRV14/Fab17 complex where the flexible CDR3 loop changed upon binding but did not induce significant conformational changes in the viral capsid [38].

### 3.2. MNV/Receptor Complex

The structure of the MNV virion/CD300lf complex showed the general location of the receptor but was of insufficient resolution to determine the contact details or the possibility of conformational changes [11]. Those details were elucidated with the atomic structure of the isolated P domain/CD300lf complex [23,39] (Figure 3B,D). The CD300lf contact site is near the top of the P2 domain, between the A’/B’ and D’/E’ loops. Interestingly, this contact surface includes some of the amino acids involved in the binding of the neutralizing antibody, A6.2. From structural analysis, Nelson et al. also found that GCDCA and lithocholic acid (LCA) bind in two deep pockets in the P domain dimer interface, distant from receptor and antibody binding sites [23].

Interestingly, the conformations of the A’/B’ and E’/F’ loops in the receptor complex [23] are nearly identical to that of the closed conformation observed in the original P domain crystal structure [31]. The major difference is that the C’/D’ loop in the receptor complex is ‘turned up’ compared to the P domain alone. This affords enough space for the bile salt, GCDCA (mauve spheres in Figure 3), to bind. It may be that the structural equilibrium in solution is shifted by GCDCA binding towards the closed form of the A’/B’ and E’/F’ loops, which may be preferred by CD300lf. In this way, bile salts may enhance receptor binding, allosterically, by altering the dynamics and structural equilibrium of the P domain. What is particularly interesting is that one of the two escape mutants to the neutralizing antibody 2D3, V339, lies immediately adjacent to the bile salt binding site. As with the bile salts, we proposed that the V339I mutation shifted the equilibrium towards the closed conformation that does not favor antibody binding [26,34,36]. It is rather interesting to consider that the V339I escape mutant and bile salts drive the P domain conformation towards the closed conformation that favors receptor binding and at the same time away from the open conformation favored by antibody binding.

What is clear from these high-resolution structures is that antibodies and CD300lf bind to opposing P domain conformations (Figure 3C,D). In Figure 3C, the structure of the Fab/P domain complex is shown in solid colors and the P domain from the CD300lf complex is shown as a transparent structure. The yellow arrow shows how the E’F’ loop from the CD300lf complex, in the closed conformation, would collide with the CDR3 loop from the antibody. Similarly, the closed conformation of the A’B’ loop is too close to the bound antibody. Figure 3D shows the CD300lf/P domain complex as solid colors and the P domain from the Fab complex as transparent ribbons. Here, the open conformation of the P domain would place the A’B’ and D’E’ loops too close to CD300lf. Together, these structures show that the antibodies bind to the open conformation while the receptor binds to the closed conformation.

These structural results suggest that conditions that shift the P domain structural equilibrium towards the closed conformation would enhance receptor binding while blocking antibody binding. Indeed, this has been shown to be the case. As reviewed above, bile salts and metal ions enhance the CD300lf binding to the P domain [23]. Bile [11], low pH [20] and metal ions [40] cause contraction of the P domain onto the shell and converts the P domain to the closed conformation, and importantly, all of those conditions block antibody binding [11,20,37]. Studies are underway to ascertain whether low pH causes a concomitant increase in receptor affinity. Nevertheless, the structural and biochemical data clearly show that antibodies bind to the open conformation while the receptor recognizes the closed conformation, and these environmental signals switch the virus between the two states.

**Figure 4:**
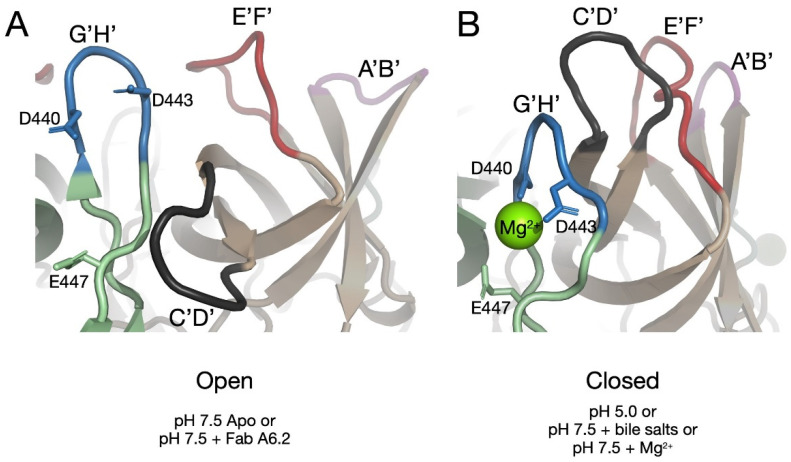
Environmentally driven changes in the G’H’ loop may control the P domain conformation. Shown here are ribbon diagrams of the tip of the P domain with the same color scheme as the previous figures. (**A**) There are three acidic groups on the G’H’ loop (D440, D443, and E447). At a pH of 7.5, these are expected to be deprotonated and therefore charged and repulsive. Indeed, under these conditions, the more vertical conformation of the loop allows the acidic sidechains to be well separated [31,37]. In this position, there is now room for the C’D’ loop to be in the downward conformation and thus allowing the A’B’/E’F’ loops to be in the open conformation. (**B**) At acidic conditions [20] or at neutral pH but in the presence of metals [40], these acidic groups are allowed to move into proximity and cause the G’H’ loop to distort, filling the space normally occupied by the C’D’ loop. This starts a chain reaction that leads to the closed conformation of the P domain.

What was not immediately clear from these results was how the addition of bile, low pH, and metals could all result in the same contracted, closed structure. Figure 4 shows the conformations of the P domain loops in the open conformation at pH 7.5 and the closed conformation at pH 5.0 or in the presence of metals or bile. In the open conformation, the G’H’ loop is in a vertical orientation. At pH 7.5, the acidic groups shown in the figure are expected to be charged and their repulsion likely drives the more vertical orientation. This opens up space that is filled by the C’D’ loop pointing in the down direction. Together, this creates space at the tip of the P domain so that the A’B’/E’F’ loops can splay apart in the open conformation. In the transition to the closed state, the G’H’ loop widens and displaces the C’D’ loop that subsequently moves to the up position. This, in turn, presses the A’B’/C’D’ loops together to form the closed conformation. Initially, we proposed that the C’D’ loop was the major trigger point in the open to closed transformation [37]; however, our more recent results suggest that the G’H’ loop may play an important role as well [20]. It is easy to envision that bile salts binding under the C’D’ loop (Figure 3B) would move it to the up position and drive the rest of the structural changes towards the closed conformation. However, it was unclear how metals and low pH could do the same if the C’D’ loop was the main driver. Shown in Figure 4B is the atomic structure of the P domain from the CD300lf complex [23]. Mg^2+^ binds to the three acidic groups on the G’H’ loop and brings them into proximity, thereby distorting the G’H’ loop. This structure is also nearly identical to the pH 5.0 structure without bile or metals being present [20]. In this case, the low pH is expected to protonate the acidic groups, and this allows them to cluster together via hydrogen bonds. In this way, via the C’D’ and G’H’ loops, these three disparate conditions (bile, pH, and metals) can all cause the P domains to shift to the closed conformation. In addition, since CD300lf binds to the closed conformation, this explains how bile and metals synergistically enhance receptor binding.

### 3.3. Bile/Low pH/Metals Rotates the A/B P Domain Dimers, Causing the P Domain Contraction

**Figure 5:**
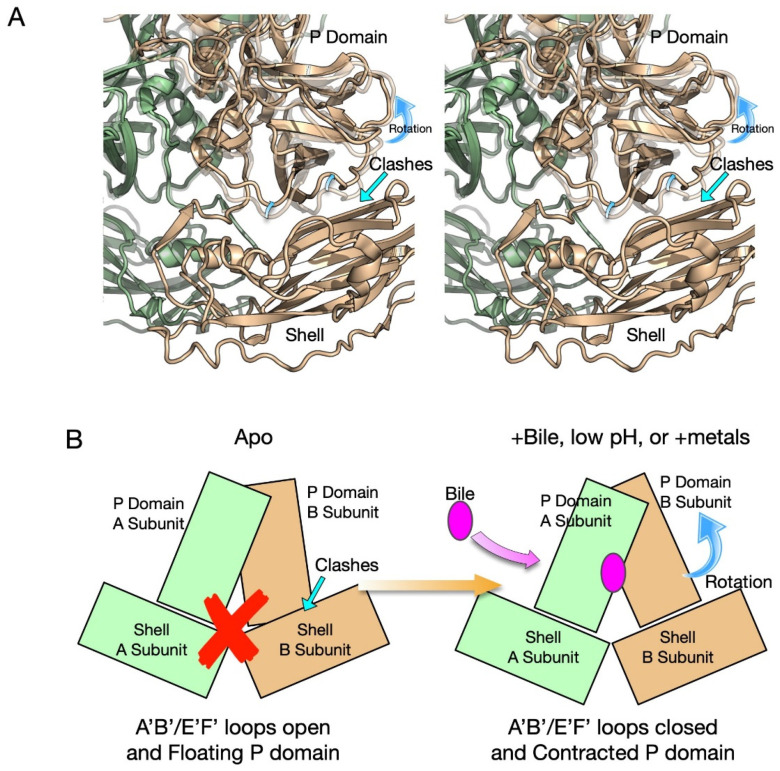
Rotation of the A/B P domains and resulting contraction onto the shell. (**Panel A**) shows a stereo ribbon diagram of the unrotated A/B dimer (open conformation) modeled onto the A/B dimer in the contracted, closed conformation observed in the low pH and bile complex structures. The open conformation (unrotated) is shown as a transparent image and the closed conformation in solid colors. Note that the P1 domains of the A subunit (light green) were used for the alignment process and therefore match well. However, this places the P1 domain of the B subunit in the open conformation too close to the shell and, hence, why the P domain in the open conformation (unrotated) cannot rest on the shell. (**Panel B**) shows a schematic representation of the structures shown in (**A**). Before the A/B subunit rotation, the surface of the P domain base is not complementary to the shell surface and the P domain is not able to rest upon the shell (red X). This changes upon rotation of the A/B subunits, where the base of the P domain dimer changes and forms a complementary surface to the top of the shell, thus allowing the P domain to rest upon the shell.

The above discussion is a model for how bile, low pH, and metals push the P domain towards the closed conformation to which the receptor binds but antibodies cannot. However, the next question is how these conditions can also cause the contraction of the P domain onto the surface of the shell [11,20,37,40]. The core of the P1 domain itself (residues 500–530) is unaffected by the conformational changes occurring in the loops in the P2 domain. Therefore, structural alignments were performed using only this region [37] when comparing the structures of the closed conformation (presence of bile, low pH, or metals) and the open conformation (pH 7.5 PBS ± Fab A6.2). If the relationship between the A and B subunits are unaffected by bile binding, then the P1 domains of the B subunits would be expected to align as well as the fitted P1 domains of the A subunits. However, what was evident from this alignment is that bile causes the A/B dimers to rotate with respect to each other [37]. When the aligned P domain structure without bile was placed onto the shell via P1 domain alignment, extensive clashes with the shell were apparent (Figure 5). Indeed, the open conformation has five times more contacts between the B subunit and the shell that are less than 3.5 Å, and 21 contacts are less than 2 Å than the closed, contracted conformation. In the closed conformation, the subunits rotate about each other and create a complimentary surface to the shell domain. This process is shown in the cartoon in Figure 5B. In the apo (pH 7.5 PBS) structure the two domains have not rotated and the surface at the base of the P domain is not complementary to the shell surface. Therefore, the P domain cannot rest upon the shell and floats above the surface of the shell. The red X denotes that the clashes prevent P domain/shell interactions. However, when the loops at the tip of the P domain change to the closed conformation, the two subunits rotate about each other and form a complementary surface with the shell, and this allows for the contraction of the P domain.

From the analysis of all structural information collected to date, this rotation/contraction seems likely governed by the movement in the C’D’ loop that, in turn, is linked to the G’H’ loop. This correlation is made evident when all the conditions and structures are compared (Table 1). In the original apo crystal structure of the isolated P domain, the A’B’/E’F’ loops were observed to be in both the open and closed conformations but the C’D’ loop was in the down position. We hypothesized that the A’B’/E’F’ loops were free to exist in both conformations without the C’D’ loop pressing against them [37], and the C’D’ loop could be in the down position only if the G’H’ loop had the vertical conformation [20]. Importantly, the A/B subunits were not rotated about each other as observed when bile, low pH, or metals are added. From EM studies of the whole virus under the same conditions, the P domain is floating above the shell and highly mobile, and therefore, the conformation of the loops in that structure is not known to high resolution. However, the crystal structure of the P domain (lines 1 and 2) and the EM structure of the P domain/Fab A6.2 under the same conditions (line 6) suggest that the C’D’ should be pointed downwards and the A/B domains are not rotated about each other. Once bile (lines 4 and 7), low pH (line 5), or metals (line 7) are added, four structural changes happen concomitantly: the G’H’ loop deforms, the C’D’ loop points upward, the A/B subunits rotate about each other, and the P domain contracts onto the virion surface. The importance of the C’D’ loop in the A/B rotation is reasonable since the C’D’ loop comprises ~400 Å^2^ of the total ~1800 Å^2^ dimer interface surface area [37], so changes in its conformation would have a large effect on the A/B interface. Simply put, the A/B subunits do not rotate about each other unless the C’D’ loop is pointing upwards, and the P domain does not contract onto the shell unless the A/B subunits have rotated. In these data, the G’H’ loop appears to be playing a role in moving the C’D’ loop. It is easy to envision that bile binding pushes the C’D’ loop into the up orientation by binding directly beneath it. However, metals and low pH appear to do the same by distorting the G’H’ loop via three acidic residues as discussed above (Figure 4). It is quite remarkable that three disparate conditions can all lead to the same series of allosteric structural changes, resulting in the contraction of the P domain onto the shell.

## 4. Summary of the Allosteric Structural Changes in the MNV Capsid

**Figure 6:**
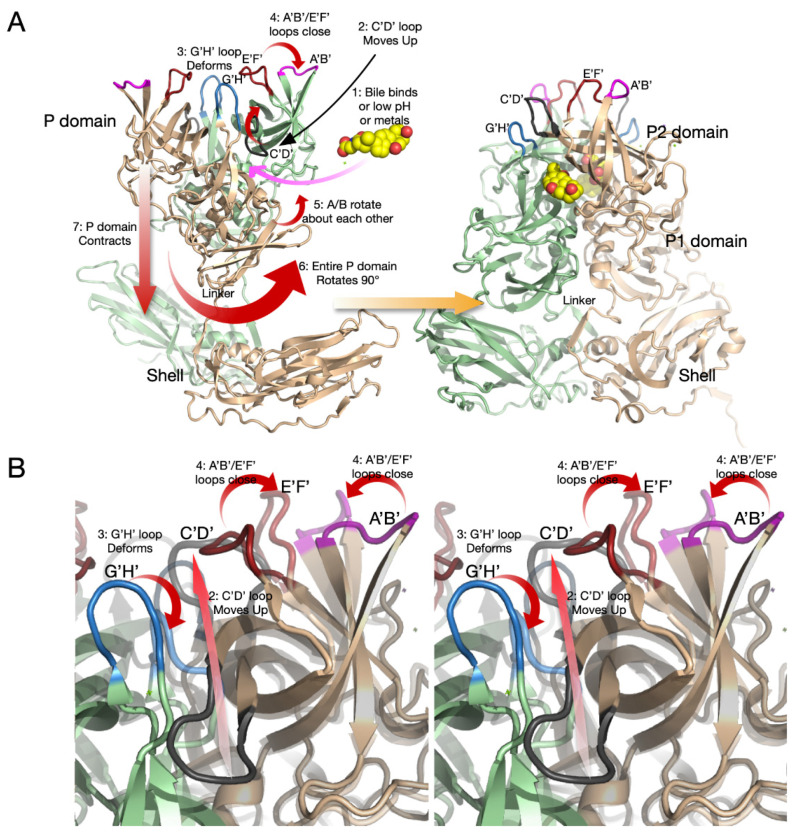
Structural details of the transition from the extended to the contracted MNV-1 states. (**Panel A**) shows the structural changes of the whole P domain transitioning from the extended to contracted structure. (**Panel B**) shows a stereo view of the concomitant conformational changes within the P domain. For clarity, the two panels are presented at different viewing angles. There are likely multiple structural equilibriums at work, and therefore, this figure represents one possible series of events. Step 1 is where three different environmental stimuli (bile, low pH, metals), alone or in concert, start the cascade of conformational changes. In step 2, the C’D’ loop moves up away from the virion surface. This process likely occurs in conjunction with step 3 where the G’H’ loop becomes distorted since they switch locations during the conformational change. When the C’D’ loop is in the up position, this leads to step 4 where the A’B’ and E’F’ loops are pushed together to the closed conformation. From our various structures, it seems likely that the movement of the C’D’ and G’H’ loops then lead to step 5 where the two subunits rotate about each other. This rotation then causes the entire P domain to rotate ~90° (step 6) and contraction of the P domain onto the shell surface (step 7). (**B**) Shown here is a stereo figure magnifying the conformational changes in the loops. The opaque ribbon diagram represents the open conformation, and the transparent figure is the closed conformation.

Figure 6A reviews the steps in the structural transition from the apo (floating P domains, open A’B’/E’F’ loops) to the contracted/closed virion structure. At a pH of 7.5, in the absence of bile and metals, the C’D’ loop is in the down position and the A’B’/E’F’ loops are splayed apart in the open conformation. We propose that since the C’D’ loop has not moved upwards under these conditions, the A/B subunits have not rotated, and therefore, the P domain cannot associate with the shell. The transformation to the contracted state starts with the addition of bile, low pH, or metals (#1). Bile may directly move the C’D’ loop upwards (#2) by binding directly beneath it. Alternatively, metals and low pH conditions may move the C’D’ loop indirectly by distorting the G’H’ loop and filling the space normally occupied by the C’D’ loop (#3). We propose that the movement of the C’D’ loop then causes the A’B’/E’F’ loops to close (#4) and to rotate the A/B subunits about each other (#5). Once the A/B subunits have rotated, the surface at the base of the P domain changes to form a complementary surface to the top of the shell. Now that the P domain can bind to the shell, the flexible linker allows the P domain to rotate by ~90° (#6) and contract onto the shell (#7). Figure 6B shows a close-up view of the loop movement described above. The apo (open) structure is shown in solid colors, while the closed conformation is shown as transparent ribbons. Again, it is important to note that this summary is constructed from several structures using both crystallographic and cryo-EM methods that all lead to the same conclusions. What is particularly interesting is that one of the two escape mutants to the neutralizing antibody, 2D3, is the buried residue V339 that lies immediately adjacent to the bile salt binding site. As with the bile salts, we proposed that the V339I mutation shifted the equilibrium towards the closed conformation that does not favor antibody binding [26,34,36]. Therefore, while mutations deep in the protein core might be expected to have a negative impact on viral viability, this allosteric escape mutant may improve receptor binding by mimicking the bile, low pH, and metal binding effects while blocking antibody binding.

## 5. Overview of Environmental Cues In-Vivo

Viruses exist on the edge of disaster; they need to be stable enough to survive movement between cells and hosts while being sufficiently unstable to respond to environmental stimuli required for infection and genome release. For example, a number of plant viruses just need the relatively slight push of neutral pH conditions and low metal concentrations to start uncoating (e.g., cucumber necrosis virus, cucumber leaf spot virus, and red clover necrotic mosaic virus [41,42]). With human rhinovirus, the virus transiently exposes its myristylated capsid N-termini in a ‘breathing’ process while waiting for the receptor to bind [43]. This delicate state is disrupted if the capsid is stabilized by the addition of hydrophobic antiviral compounds [43] or destabilized by acidic conditions.

**Figure 7:**
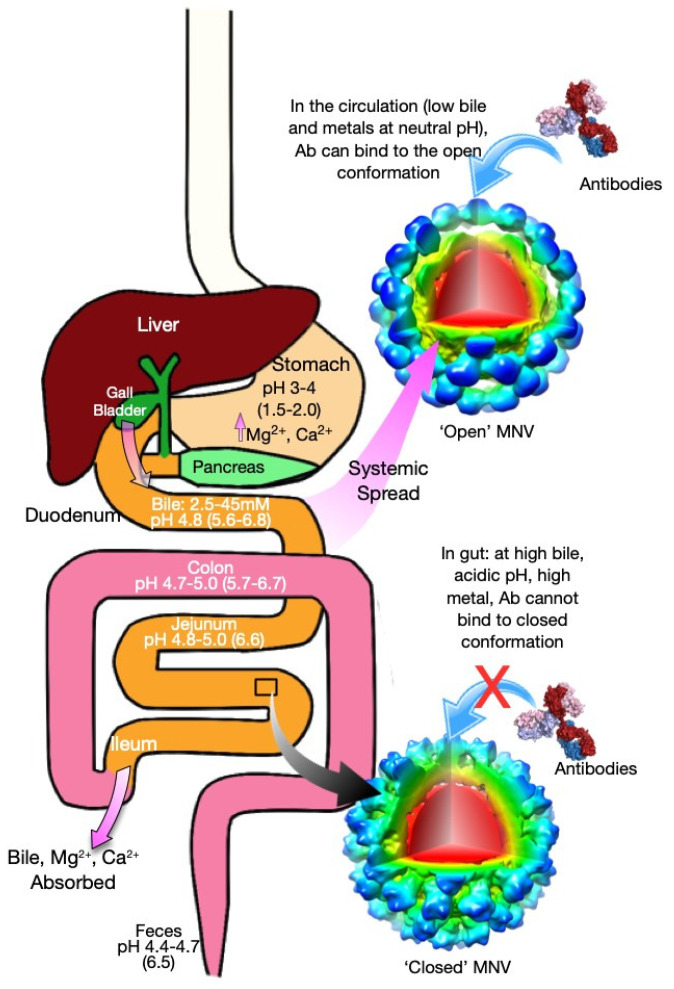
Schematic of the various states of MNV as it travels through the alimentary canal. Where available, the numbers cited are for the murine where the parenthetical values are for humans for comparison. There are three signals that cause the contraction of the P domain onto the shell and the transition of the P domain loops to the closed conformation: low pH, binding of bile salts, and the binding of metals. These conditions are found throughout the alimentary canal. In the stomach, the low pH not only causes contraction but also solubilizes ingested metal salts. In the duodenum, the pH increases to 4.8 [44], but large amounts of bile salts are deposited via the gall bladder. Here the concentration comes from measurements on humans but the flux of bile in mice is significantly greater [47]. Throughout the small intestine, the pH remains acidic, and the metal and bile concentrations remain high. In the ileum, most of the bile salts and metals are absorbed. Finally, in the colon, there are still significant metal concentrations in the ascending colon and the pH remains significantly acidic. Interestingly, the pH of the feces is also rather acidic. Therefore, the conditions throughout the alimentary canal favor the contracted, closed conformation of MNV that blocks antibody binding while enhancing receptor binding. This reverses once the infection spreads outside the intestine where the neutral pH, low metal, and low bile concentrations favor the extended conformation with the open conformation in the P domain that favors antibody over receptor binding. Not drawn to scale.

MNV differs from these viruses in that it uses at least three cues to alter its structure in response to the changing environment: pH, bile, cationic metals. Since MNV is an enteric virus, it needs to adapt to wildly changing conditions in the gastrointestinal tract. (Figure 7). In this schematic, the human form is used because of familiarity and because some similar processes may yet be identified in human noroviruses. The values for humans are shown in parentheses for comparison. MNV is likely in the contracted, closed state as it passes from the stomach through to the jejunum where the pH’s start at ~3–4 and stay fairly acidic (4.0–5.0) all the way to the feces [44]. In humans, the pH of the stomach is lower than in mice but remain higher throughout the rest of the alimentary canal. Interestingly, there are several possible trypsin cleavage sites (i.e., lysine and arginine residues) in the extended, flexible linker. Therefore, it is tempting to speculate that MNV, much like a turtle, contracts at low pH to protect itself from denaturation and digestion. In the stomach, the acidic conditions also increase metal solubility (e.g., [45]) that will both enhance receptor binding [23] and will act synergistically with the low pH to induce contraction of the P domain onto the surface. MNV then enters the duodenum where it is exposed to all three conditions that cause contraction of the P domain and enhanced receptor binding. The common bile duct connects at the duodenum and bile concentrations in the early gut are 2.5–45 mM in humans [46]. Mice have a continuous flow of bile of ~80–120 mL/kg/day, whereas the secretion of bile in humans is synchronized meal consumption and ~5 mL/kg/day [44]. The concentrations of bile and metals remain high through the small intestine and the pH remains acidic at pH 4.7–5.0, whereas it is more alkaline in humans with a pH range of 5.6–6.6. Most of the metals and bile are absorbed in the ileum with much of the remaining metal absorbed in the colon. In the murine, the colon is acidic with a pH of 4.7–5.0, whereas it is more alkaline in humans with a pH range of 5.7–6.7. Therefore, the structure of MNV is likely to remain in the close, contracted conformation throughout the alimentary canal—one that favors receptor binding while blocking antibody binding. Interestingly, the pH of murine feces is also acidic (4.4–4.7), and it is tempting to speculate that the acidic conditions might facilitate fecal/oral transfer.

Once the viral infection spreads from the gut lumen to the serum, the pH is neutral, and the bile and metal concentrations drop to low levels. Under these conditions, the P domain rises off the shell and the apical loops change to the open conformation. In this way, MNV uses all the environmental cues in the gut to simultaneously protect itself from digestion and denaturation, block antibody binding, and enhance cell binding. Once the infection spreads outside the gut, the virus adopts the expanded (floating) P domain conformation with the loops at the tip of the P domain splayed out in the open conformation.

It is interesting to note that all of the MNV monoclonal antibodies raised from infected mice that we have studied to date [12,26,31,33,34,36,37] clearly recognize the ‘open’ conformation at the apical loops observed in the apo structure at neutral pH. Importantly, the conditions used in the ELISA assays when screening the hybridomas favored the ‘open’ conformation. Recognizing that the MNV structure is in a structural equilibrium between the open and closed conformations, it is possible that there may have been hybridomas that made antibodies to the closed conformation but were not selected during the screening. Therefore, we cannot say that the mice did not produce any antibodies to the closed conformation. Future screens would be needed to investigate this possibility. Regarding the fact that the receptor apparently binds to the closed conformation, it is important to point out that MNV can bind to receptor and infect tissue outside of the gut and, indeed, MNV causes systemic infection. This might be either due to receptor binding to capsids in both the open and closed conformations or that the P domain is in both conformations in the serum, in some proportion. Bile and metal enhance receptor binding to MNV but are not absolutely required for virus/receptor interactions. SPR studies using isolated CD300lf and P domains showed weak, but measurable, interactions. Calcium improved that ~10 fold and the combination of bile and calcium acted synergistically to further enhance binding [23]. Therefore, it is more accurate to say that the conditions in the gut enhance receptor binding while decreasing the ability of antibodies to bind. While not absolute, these levels of enhanced cell attachment and antibody blockade are sufficient to drive the evolution of these structural ‘switches’.

## 6. Conclusions

These studies have shown that the calicivirus capsid is not merely a static container moving viral genome from host to host. What we have seen is that MNV has two modes of flexibility, each with different biological purposes. In the first mode, the entire P domain changes from being loosely tethered to the shell to being contracted to the shell surface. While the reversible contraction of the P domain onto the shell remains to be shown in other noroviruses, the tethered, loose association with the shell is a common feature [26,27]. The biological role for such marked flexibility of the entire P domain is still uncertain and under study. From our bile/MNV structure, bile causes contraction of the virus [11] while improving the ability of the virus to bind to the cell [23]. This seems somewhat counterintuitive since one might think that increased mobility would facilitate virus/cell interactions. However, the MNV/CD300lf models [11,23] suggest that CD300lf molecules are expected to be crowded together in the expanded capsid. It is also possible that the flexible P domain might confuse the immune system by exposing buried epitopes in the serum that are not available in the gut. Similarly, proteases may cleave the linker region when in the expanded state that would expose epitopes normally buried. Further studies are clearly needed to tease out the biological role of this extreme P domain movement from the structural changes occurring within the P domain. While this simplistic model needs extensive testing, it suggests that the P domain in solution samples many conformations and configurations, waiting for environmental cues when at the best location in the host for infection. It is fascinating that apparently the predominant structure presented to, or recognized by, the immune system is the open conformation while the conformation necessary for receptor binding is closed.

The most unexpected result from these studies is that the conformational changes occurring within the P domain appear to drive the contraction of the capsid and most biological functions. We have shown that bile, low pH, and metals rotate the P domains within the spike dimers to create a complementary surface between the P domain and the shell that leads to contraction onto the shell. This rotation, in turn, appears to be related to the C’D’ loop movement [37] that can be controlled by distortion of the G’H’ loop. In addition, all these environmental signals cause the A’B’/E’F’ loops to tightly associate into the closed conformation. This facilitates receptor binding while blocking antibody binding.

It is important to note that these results show that conversion of the P domain to the closed conformation and the resulting rotation of the P domains about each other drives the contraction of the P domain onto the shell rather than the other way around. As summarized in Table 1, even in the isolated P domain the rotation of the A/B domains does not occur unless the C’D’ loop is in the up position and the contraction of the P domain onto the surface of the virion does not occur unless the A/B domains have rotated. Therefore, the role of the conformational changes within the P domain are clear, but the biological significance of the contraction of the P domain onto the surface is not.

In summary, MNV has evolved a remarkable strategy to infect the host and evade the immune system. MNV enters the alimentary canal with its target being the Peyer’s patches in the gut. It uses the extreme conditions in the gut to contract into a tightly packed shell, protecting itself against protease digestion and low pH denaturation. These same conditions alter the P domain structure itself to bind better to the receptor and the target tissue. Once it replicates and escapes into the lamina propria, the neutral pH, low metal, and low bile concentration cause the P domains to rise off the shell and the apical loops spread open. There, the B cells are trained to what they see, the open conformation. Antibodies raised against this serum form of the virus are of little use in protecting the gut against infection.

Perhaps similar processes are involved in human noroviruses, and these may be the reason for the relatively short-lived protection against subsequent infections (e.g., [48]). There a number of differences and similarities between MNV and the human genotypes. They both have flexible P domains that respond to environmental cues. While both bind to quite different receptors, the MNV/receptor interactions are enhanced by bile [23] and bile enhances HBGA binding in certain genotypes [49]. Antibodies to both have been found to recognize epitopes buried in the contracted state [27,33]. Finally, the alimentary canals of human and mice differ in significant ways (Figure 7), and therefore, the details of and biological roles of these conformational transitions are likely to differ as well.

MNV uses metabolites and environmental cues to create one shape in the gut and a completely different one to the immune system in the serum. Recent studies on SARS-CoV-2 suggest that other viruses might also use metabolites to thwart antibody binding [50]. Specifically, the authors demonstrated that heme metabolites can allosterically abrogate neutralizing antibody binding. Clearly, better understanding the structure of viruses at the site of infection versus the structure presented to the adaptive immune system will be critical in the development of more efficacious vaccines to a potentially wide range of viruses.

## Figures and Tables

**Table 1:** Summary of MNV structures in various conditions. In this table, ‘PD’ denotes that the structure was of the isolated P domain, while ‘virus’ is used when the whole virus structure was determined. For the experiments using the whole virion, it is noted whether the P domains are in the ‘expanded’ (floating) or ‘contracted’ (affixed to the shell) states. Also noted is whether the A’B’/E’F’ loops are splayed apart (open) or tightly associated (closed). Also noted is whether the C’D’ loop is in the up or down position. The second part of the table denotes how the structures were determined, the resolution, and the associated database information. NA in this table is ‘not applicable’ since the structure was of the isolated P domain and not the whole virus. ND is ‘not determined’ because the structure was of low resolution.

	Sample	Conditions	Virion	A’B’/E’F’	C’D’	A/B Rotation
1	PD	pH 7.5	NA	Open	Down	No
2	PD	pH 7.5	NA	Closed	Down	No
3	Virus	pH 7.2	Floating	ND	ND	ND
4	Virus/bile	pH 7.2	Contracted	Closed	Up	Yes
5	Virus	pH 5.0	Contracted	Closed	Up	Yes
6	PD/Fab	pH 7.4	NA	Open	Down	No
7	PD/bile/CD300lf	pH 8.7, metals	NA	Closed	Up	Yes
8	Virus	pH 7.6, metals	Contracted	Closed	Up	Yes
	**Method**	**Resolution**	**PDB/EMDB**		**Citation**	
1	X-ray	2.0 Å	3LQ6		[31]	
2	X-ray	2.0 Å	3LQ6		[31]	
3	EM	8.0 Å	/7564		[12,26]	
4	EM	3.1 Å	/20250		[11]	
5	EM	3.3 Å	7N6Y/24211		[20]	
6	EM	3.2 Å	7L5J/23187		[37]	
7	X-ray	2.0 Å	6E47		[23,39]	
8	EM	3.1 Å	6S6L/10103		[40]	
